# Victories

**Published:** 1873-02

**Authors:** W. C. Barrett


### Victories.

W. C. BARRETT, M. D. S.

Our great triumphs are made up of little victories, and I propose to relate some of mine, and thus attempt by small installments to repay some of my indebtedness for the simply narrated incidents of office practice, which I have often read and profited by. Of course no claim of originality of procedure is advanced; yet perhaps by combining the ideas of others I may in some cases arrive at results, which shall be suggestive of something useful.

Some of our religious denominations hold social meetings at which each in turn gives an epitome of his trials, his temptations, his struggles, and his victories. Why may not the readers of the REGISTER hold such an "experience meeting." Surely the simple narrative of some of our combats, and the consequent victories or defeats may be both entertaining and instructive. Therefore, "I arise to explain."

I lately had occasion to take a lower impression for an old lady of sixty-five. The lower teeth were all out, but in the upper jaw were four or five bicuspids and molars. The lower maxilla was large and broad, while the oral aperture was exceedingly small, with tense, rigid *orbicularis oris.* I could not introduce an ordinary impression cup without great difficulty, and when I did so a fold of the buccal mucus membrane would intervene and spoil the result. Finally I got the best wax impression possible, and from this drew a plaster cast. Over this cast I moulded a cup of thin rolled tin, and upon its convex surface I soldered a thicker piece to stiffen it, allowing a projection for a handle. Then with a very little thin plaster of Paris I succeeded with no difficulty in getting a capital impression; a thing impossible to obtain in any case with a poorly adapted cup. I dare say, that in attempting with an ordinary cup to get an impression of some difficult case, I have often spent sufficient time to have made half a dozen, which should be exactly adapted to the mouth, and which would have given me a perfect impression at the first trial.

Miss D—— had a large cavity in the posterior approximal surface of an inferior first molar. The teeth were large, firm, in close contact, and were all present. The cavity was low down, and extended beneath the margin of the gum. The perplexities to be encountered were a lack of space, and the difficulty of keeping the cavity dry. Neither a ligature nor the clamps would hold the rubber dam down sufficiently low to prevent the ingress of blood, and a wedge would be too much in my way. I cogitated till a brilliant thought struct me. I had some of Jack's Matrices, but had never used them, and here was a capital chance to test their virtues. Nothing else that I could think of would exactly fill the bill, and if these would, why, I was armed with a new weapon. I separated the teeth with a No. 1 Froid file, and with my burring engine proceeded to open from the crown down to the cavity. When this was thoroughly done I slipped on the rubber dam, crowded a matrix down between the teeth till it had forced the rubber past the cervical edge of the cavity, wedged the matrix fast, and after finishing the excavating, proceeded to fill *as a simple crown cavity*, with, as Mark Twain would say, "that unflinching faith a Christian feels in four aces." When I was through, and had removed the matrix, I was somewhat surprised to find, that except upon the crown, my filling needed no finishing. Its proximal surface had no protruding edges, and was as good as I could make it. Since that time I have kept Jack's matrices in a convenient place not to use for every approximal cavity, but wherever they are indicated. Whenever a weapon has proved particularly useful in any encounter we are, I think, too apt to throw away all our other arms, and attempt to rely wholly upon that, whereas, it is perhaps only serviceable in those cases in which other weapons fail. The matrix is to me a powerful auxillary, but not my sole dependence.

We sometimes find it difficult to force the rubber dam between teeth that are in close contact. If a little soap (fine toilet of course) be wet, and rubbed upon that surface of the dam, which first comes in contact with the teeth, it is astonishing how easily it slips through. A friend first suggested this to me. He obtained it at second hand, yet it is fully warranted to be as efficacious as though originally discovered by the reader of this article. Strange to say the idea has not as yet been patented, and I give it this publicity to head off some prospective caveat.

From original defects, and the subsequent action of caries, Mrs. R—— had lost nearly the upper half of the crown of an inferior central incisor, causing great annoyance, not only from the disfigurement and imperfect articulation but from the difficulty of retaining fluids in the oral cavity. As she expressed it, "Her mouth leaked, she lisped, and besides she looked bad." The case presented these difficulties. The top of the remnant was nearly a level surface, and yet the tooth was so small that a restoration to the desired length would not be sufficiently strong, if only anchored by retaining pits upon its surface. Mack's screws were impracticable for the same reason—the extreme smallness of the tooth. Finally, after adjusting the rubber dam, I drilled a pit with my burring engine near the gum in each proximal surface, as deep as I thought consistent with the strength and vitality of the tooth. Then from the surface of the crown I excavated a furrow down each side of the tooth, till it communicated with the pit near the gingival margin; which pit was made slightly retaining in shape. A transversed stria across the top united the two. I commenced the filling at the pits, carrying it up to the surface of the crown on either side, and by means of the broader trench across the surface the two golden legs of my filling were united. To the broad, transverse bond, No.

60 annealed gold was securely welded, and thus the crown gradually built up. It cannot now be disturbed without either breaking both the golden legs or straps which support it, and which from the inherent tenacity of the metal is quite improbable; or by fracturing the tooth. I avoided the weakening of the latter by not drilling the pits upon the same line, but the one above the other.

